# Successful use of Proprotein Convertase Subtilisin/Kexin Type 9 Inhibitors in Hypertriglyceridemia-induced Acute Pancreatitis: A Case Report

**DOI:** 10.2174/011573403X343784241115055037

**Published:** 2025-01-03

**Authors:** Rundi Qi, Hailei Liu, Xin Li, Minglong Chen

**Affiliations:** 1 Department of Cardiology, The First Affiliated Hospital of Nanjing Medical University, Nanjing, China;; 2 Department of Cardiology, Nanjing BenQ Medical Center, The Affiliated BenQ Hospital of Nanjing Medical University, Nanjing, Jiangsu Province, China

**Keywords:** Hypertriglyceridemia, acute pancreatitis, proprotein convertase subtilisin/kexin type 9 inhibitor, triglyceride reduction, low-density lipoprotein cholesterol, acute abdominal pain

## Abstract

**Introduction:**

Managing hypertriglyceridemia-induced acute pancreatitis (HTG-AP) can be challenging, particularly due to the need for rapid triglyceride reduction to below 500mg/dL (5.645 mmol/L).

**Case Report:**

This is a case describing a 39-year-old female patient who presented to the Emergency Department with acute abdominal pain resulting from severe HTG-AP. However, under conventional therapy with oral lipid-lowering drugs, the triglyceride levels remained uncontrolled. Oral moderate-intensity statins could not only reduce low-density lipoprotein cholesterol (LDLc) by 25%-50%. However, increasing the dose could not further reduce blood lipids while increasing the risk of liver damage. After the administration of proprotein convertase subtilisin/kexin type 9 inhibitor (PCSK9i), the triglyceride levels were well controlled with no additional side effects, and the symptoms of the patients were completely relieved.

**Conclusion:**

In cases of unsatisfactory lipid control under conventional therapy, PCSK9i may offer a viable option for managing HTG-AP.

## 1 INTRODUCTION

Acute pancreatitis (AP) is the most common acute gastrointestinal disease, affecting multiple organs and several systems. AP can progress into severe acute pancreatitis (SAP) with high fatality [1, 2]. Gallstones and chronic alcohol abuse remain the leading causes of AP. Hypertriglyceridemia is the third most common cause globally. Hyperlipidemia has surpassed alcohol as the second leading cause of AP in China nowadays. The prevalence of hypertriglyceridemia-induced pancreatitis (HTGP) has been reported to be as high as 22%. However, it is generally thought to account for approximately 5% of all cases of AP and up to 56% of AP cases during pregnancy [3-7]. Hyperlipidemia-induced AP is generally associated with a significant increase in serum triglyceride levels, namely hypertriglyceridemia induced AP (HTG-AP). The pathophysiology of HTG-AP is associated with the accumulation of free fatty acids (FFA), pancreatic microcirculation disorder, and the activation of the inflammatory response. Triglycerides are not inherently toxic to the pancreas, but it is the breakdown of triglycerides into FFAs by pancreatic lipase that causes lipotoxicity. The severity of pancreatitis is dependent on the severity of the inflammatory response and the injury caused by lipotoxicity. The management of HTG-AP is complicated, with etiological treatment playing an important role [8-11]. Besides conventional treatment for HTG-AP, including insulin, plasmapheresis, and fibrates, a rapid drop of serum triglyceride levels to 500 mg/dL (5.645 mmol/L) is pivotal. Various cholesterol-lowering drugs, including statins, ezetimibe, nicotinic acid, bile acid sequestrants, proprotein convertase subtilisin/kexin type 9 inhibitors (PCSK9i), niacin, fibrates and n-3 fatty acids (*e.g*., icosapent ethyl), are available [12-15].

PCSK9i, utilizing human monoclonal antibodies, is based on the following mechanisms. PCSK9 forms a bond with low-density lipoprotein receptor (LDLR) and promotes its breakdown by preventing LDLR from migrating back to the cell surface. PCSK9 increases triglyceridemia (TG) by consuming LDLR and preventing the metabolism of intermediate-density lipoprotein and chylomicron (CM) residues. It may also increase TG by degradation of other LDLR-like receptors, such as CD36 and VLDL receptors [10, 16]. PCSK9 inhibitors reduce the level of LDL-C in circulation mainly by blocking the binding of PCSK9 and LDL receptor (LDLR) in circulation, thereby preventing the degradation of the PCSK9-LDLR complex through internalization and promoting the metabolism of LDL-C [17]. It has been approved for the second-line treatment of high cholesterol in adults. PCSK9i is also an alternative for patients with statin intolerance, high cardiovascular risk, and a lack of efficacy of conventional therapy. In a systematic review, McDonagh *et al.* reported that the use of a PCSK9i (alirocumab or evolocumab) resulted in a greater reduction in low-density lipoprotein cholesterol in patients with HTG who were initially on high-intensity statins plus ezetimibe, with a greater improvement in high-density lipoprotein cholesterol (HDL-C) [18]. However, the role of PCSK9i in patients with HTG-AP remained largely unknown. We herein present a case of HTG-AP-managed PCSK9i in addition to traditional therapy.

## 2 CASE STUDY

**Fig. (1):**
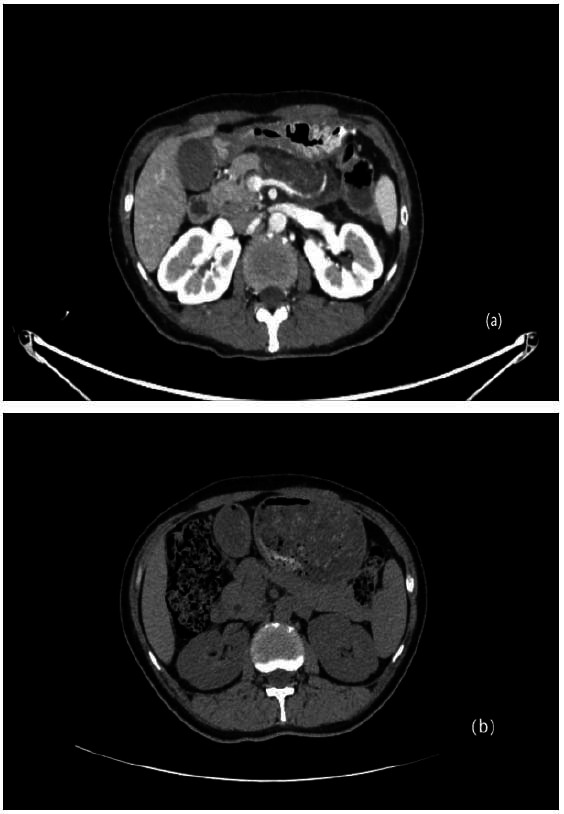
CT scan of the patient’s abdomen (patient identifiers removed). (**a**) CT scan indicated an AP with a full and plump shape of the pancreatic head on the first day of the HTG-AP. (**b**) CT scan indicated a normal pancreas after a month of the HTG-AP.

A 39-year-old female presented to the emergency department with aggravated abdominal pain for 10 hours (visual analogue scale score: 10/10) after lunch. No history of hyperlipidaemia or family history of hyperlipidaemia was reported. The patient was alert, with vital signs including a blood pressure of 152/60 mmHg, a pulse rate of 71 beats/min, a body temperature of 36.2°C, and an oxygen saturation of 98% under ambient air. Upon clinical examination, her abdomen was palpated with epigastric tenderness. Laboratory tests revealed extremely high triglyceride levels of 9480.2 mg/dL (107 mmol/L), increased serum amylase levels of 1150 U/L, an increased leukocyte count of 11, 030 cells/uL, a neutrophil ratio of 85.8%, a c-reactive protein of 11.5 mg/L and a glycosylated haemoglobin of 13.2%. An abdominal computed tomography (CT) scan indicated an AP with a full and plump shape of the pancreatic head (Fig. **1a**). During the follow-up, a CT scan indicated a normal pancreas after a month of the HTG-AP (Fig. **1b**). She had no history of biliary tract disease, alcohol, trauma, or tumor. Routine treatments, including abstinence from food, active fluid infusion with 3500 ml per day, acid inhibition with lansoprazole 30 mg intravenously twice a day, nutritional support including potassium 2.5 g, vitamins 40ml, and Sodium Lactate Ringer's Injection 500 ml intravenously per day, pain management with flurbiprofen axetil injection 50 mg intravenous injection while in pain and blood glucose management with insulin, were implemented. On the third day, TG was not well controlled. Serum amylase levels decreased to 149 U/L, but the symptoms remained. Therefore, PCSK9i (tafolecimab, 150 mg, Innovent) was applied subcutaneously. On the seventh day, her TG levels decreased to 4.99 mmol/L (442.1 mg/dL), serum amylase levels decreased to normal, which is 30 U/L, and her abdominal pain symptoms were relieved. On the eighth day, the patient resumed eating. We recommended that the patient be in a healthy dietary mode. After 10 days of hospitalization, her symptoms were completely relieved, and she was discharged. On the eighteenth day, she received a second injection of tafolecimab. Then, PCSK9i was regularly administered every two weeks. On the 41^st^, 80^th^, and 108^th^ days, blood tests were performed, and the trend in blood lipid levels is shown in Fig. (**2**). The blood lipids decreased to normal and maintained normal levels effectively. The trend in serum amylase levels is shown in Fig. (**3**). The study protocol adhered to the principles of the Declaration of Helsinki and was approved by the Institutional Review Board of the Affiliated BenQ Hospital of Nanjing Medical University. The ethics committee approval number is 2024-KL004. The patient had provided written informed consent.

## 3 DISCUSSION

To the best of our knowledge, this is the first case to introduce the application of PCSK9i in patients with HTG-AP and to determine the potential role of PCSK9i in patients with HTG-AP, especially in the setting of failed conventional therapy.

AP and hypertriglyceridemia mutually interact. Patients with HTG have a high (14%) incidence of acute pancreatitis and a greater mortality than patients with other precipitating factors [19, 20]. Previous studies have indicated that elevated serum triglyceride levels are independently associated with a more severe course of pancreatitis [21]. According to the 2012 American Classification of AP [22], this patient met the diagnostic criteria for HTG-AP. The possible mechanisms of PCSK9i to reduce TG levels are summarised as follows: Serum PCSK9 concentration affects the metabolism of TG-rich lipoproteins both in the intestine and liver origin. In fact, in most studies, PCSK9 levels are modestly correlated with TG levels. PCSK9 inhibitors block the binding of PCSK9 and LDLR in circulation directly, thereby preventing the degradation of the PCSK9-LDLR complex through internalization. Additionally, a decreased secretion of TG-rich lipoproteins has also been suggested to participate in the PCSK9i-associated decrease of TG levels. In fact, LDLR directly affects apoB stability through enhanced autophagic degradation in hepatocytes. Thus, PCSK9 inhibitors associated with increased LDLR expression lead to an increased degradation of apoB, resulting in a reduced synthesis of apoB-containing lipoproteins. [23]. In addition to the LDL receptor, PCSK9 degrades the VLDL receptor, the Apo E receptors ApoER and ApoER2, the cluster of differentiation 36 and 81 (CD36 & CD81, respectively), beta-secretase 1 (BACE1), and the epithelial (NA+) channel (ENaC). The VLDL receptor is essential for PCSK9-dependent regulation of fatty acid uptake in different tissues [16]. ApoER is critical for lipoprotein metabolism and cell motility. In addition to its role in lipid metabolism, PCSK9 is also expressed in pancreatic insulin-secreting beta cells and has been shown to play a part in normal insulin homeostasis, which is also useful for HTG-AP management [24-27].

The initial treatment of HTG-AP, including fluid resuscitation, pain control, and nutritional support, is consistent regardless of the cause. Prompt recognition of HTG as a cause of acute pancreatitis is pivotal for facilitating the timely management of this disease to prevent recurrent acute pancreatitis. Once HTG-AP is diagnosed, all measures should be considered to control both short-term and long-term lipid levels with the goal of maintaining triglyceride levels at less than 5.645mmol/L [19, 28]. The ELEFANT trial demonstrated that early elimination of HTG and toxic free fatty acids (FFAs) can reduce the risk of mortality and the severity of HTG-AP [29]. Given that PCSK9i is expensive and may not be widely available, for those who cannot be well controlled or who have adverse reactions to conventional oral lipid-lowering drugs, PCSK9i can be used as a substitute or added as a supplement.

Nonetheless, the study was based on a single case report. Thus, larger studies to confirm the applicability of PCSK9i in HTG-AP management would provide a further full perspective.

## CONCLUSION

Thus, triglyceride content is pivotal for HTG-AP, and the combination of PCSK9i could be considered in the setting of unsatisfactory lipid control under conventional treatment. Long-term studies on the safety and efficacy of PCSK9i in this population are warranted.

## Figures and Tables

**Fig. (2):**
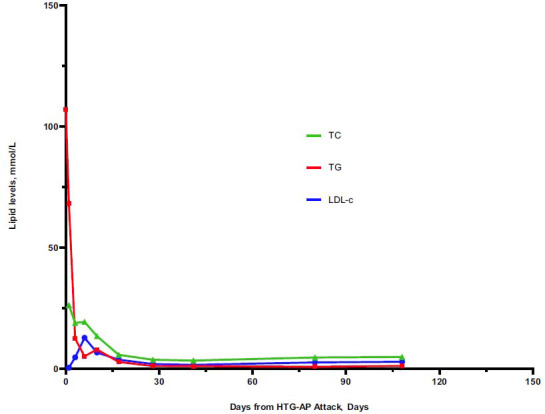
The lipid levels trend of the patient from the days of HTG-AP attack. **Abbreviations:** TC, total cholesterol; TG, total triglycerides; LDL-c, low-density lipoprotein cholesterol.

**Fig. (3):**
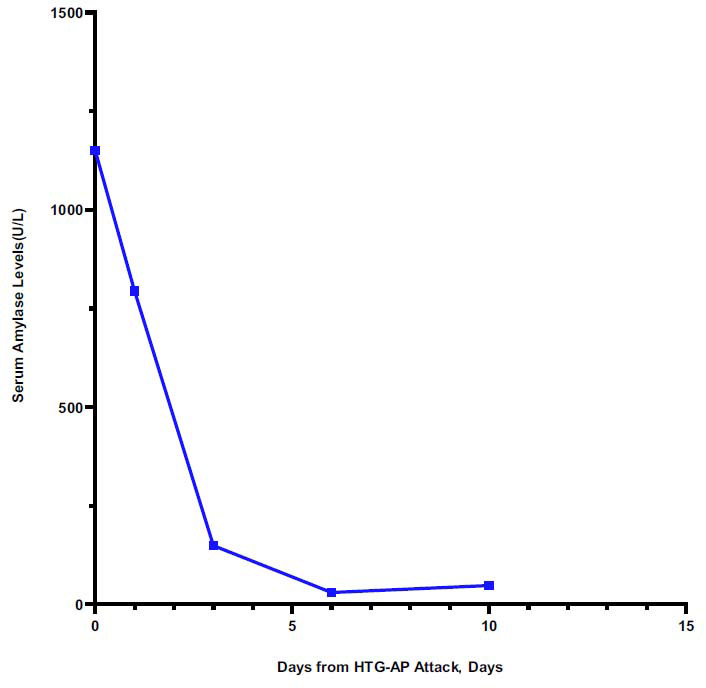
The serum amylase levels trend of the patient from the days of HTG-AP attack.

## Data Availability

All data generated or analyzed during this study are included in this published article.

## LIST OF ABBREVIATIONS

SAP: Severe Acute Pancreatitis
HTG-AP: Hypertriglyceridemia Induced AP
PCSK9i: Proprotein Convertase Subtilisin/kexin type 9 Inhibitors
LDLR: Low-density Lipoprotein Receptor
TG: Triglyceridemia
CM: Chylomicron
FFA: Free Fatty Acids
HDL-C: High-density Lipoprotein Cholesterol
